# Potential benefits of *Cuminum cyminum L* supplementation on components of metabolic syndrome in adults with metabolic disorders: a GRADE-assessed systematic review and meta-analysis of randomized controlled trials

**DOI:** 10.3389/fnut.2025.1618108

**Published:** 2026-01-13

**Authors:** Man Liu, Songze Wu, Maryam Falahatzadeh

**Affiliations:** 1 Department of Cardiology CCU, West China Hospital, Chengdu, China; 2 Department of Respiratory and Critical Care Medicine, West China Hospital, Chengdu, China; 3 Department of Pharmacy, Shiraz University of Medical Sciences, Shiraz, Iran

**Keywords:** *Cuminum*, lipids, triglycerides, metabolic syndrome, meta-analysis

## Abstract

**Introduction:**

*Cumin* (*Cuminum cyminum* L.) is recognized for its anti-diabetic, anti-inflammatory, and antioxidant properties, which may influence components of metabolic syndrome (MetS) in adults with metabolic disorders. However, previous studies have reported inconclusive and sometimes conflicting results. Therefore, this meta-analysis aimed to evaluate the effects of *cumin* supplementation on MetS components in adults with metabolic disorders.

**Methods:**

A systematic search of electronic databases, including Web of Science, Scopus, Embase, and PubMed, was conducted up to April 2025 to identify randomized controlled trials (RCTs) evaluating *cumin* supplementation in adults with metabolic disorders. Subgroup and sensitivity analyses were performed to explore heterogeneity. The search was restricted to studies published in English. Publication bias was assessed using Begg’s test.

**Results:**

A total of nine studies published between 2013 and 2020, with intervention durations ranging from 8 to 24 weeks, were included in the meta-analysis. The results indicate that *cumin* supplementation significantly affected several components of MetS. Specifically, *cumin* demonstrated a significant reduction in fasting blood sugar (FBS) (SMD: −1.38; 95% CI: −2.26 to −0.50, *p* = 0.002; *I*^2^ = 94.6%, *p* < 0.001), triglycerides (TG) (SMD: −0.58; 95% CI: −1.14, −0.02, *p* = 0.044; *I*^2^ = 88.7%, *p* < 0.001), and waist circumference (WC) (SMD: −0.46; 95% CI: −0.87, −0.04, *p* = 0.033; *I*^2^ = 56.1%, *p* = 0.077). Additionally, *cumin* was associated with a significant increase in high-density lipoprotein cholesterol (HDL-C) (SMD: 0.77; 95% CI: 0.02 to 1.52, *p* = 0.045; *I*^2^ = 92.3%, *p* < 0.001). Furthermore, subgroup analysis revealed that *cumin* could have more beneficial effects in older adults (>50 years old) in term of FBS and TG. Also, lower doses of *cumin* supplementation were responsive for improvement in FBS and HDL-C levels.

**Conclusion:**

This meta-analysis suggests that *cumin* supplementation may have beneficial effects on several components of MetS, including FBS, lipid profile (TG and HDL-C), and WC, in adults with metabolic disorders. However, further high-quality, large-scale trials are needed to strengthen the evidence and confirm these findings.

## Graphical abstract

**Figure:**
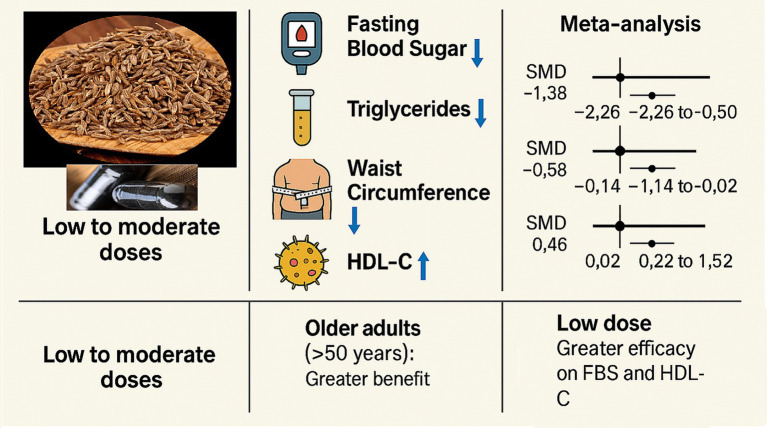

## 1 Introduction

Metabolic syndrome (MetS) is a complex condition influenced by aging, genetics, and lifestyle factors such as physical inactivity, poor nutrition, and obesity, contributing to its increasing global prevalence (1, 2). MetS shares many pathophysiological features with type 2 diabetes (T2DM), obesity, prediabetes, and non-alcoholic steatohepatitis (NASH), including insulin resistance, dyslipidemia, and chronic low-grade inflammation (3). Epidemiological studies estimated that the incidence of MetS will reach 53% by the year 2035 (4). The most widely used diagnostic criteria for MetS are provided by the National Cholesterol Education Program Adult Treatment Panel III (NCEP-ATP III) and the International Diabetes Federation (IDF) (5, 6). These criteria include elevated fasting blood sugar (FBS), blood pressure (BP), triglycerides (TG), waist circumference (WC) (central obesity) and reduced high-density lipoprotein cholesterol (HDL-C) (7). Likewise, it is crucial to halt its growing prevalence using preventive strategies. In this context, *Cumin* (*Cuminum cyminum L*.) has been demonstrated to be effective in metabolic panel.

*Cumin* is a medicinal plant from the family Apiaceae, native to the Eastern Mediterranean and South Asia (8). The dried seeds of *cumin* are used as a culinary spice in many countries (9). *Cumin* seeds contain over 100 different chemicals, including fixed oil, volatile oils, essential fatty acids, protein, vitamins and minerals. Previous research has shown that this spice has anti-inflammatory, antioxidant, anti-diabetic, and immune-modulating activities (10, 11). Anti-inflammatory effects are mediated with downregulation of pro-inflammatory cytokines [e.g., tumor necrosis factor-α (TNF-α), interleukin-6 (IL-6)] (12). In addition, bioactive compounds found in *cumin* have been shown to mitigate the oxidative stress by neutralizing free radicals (9, 13). Enhanced insulin secretion following cumin supplementation supports its insulin-sensitizing properties (14). Some studies also suggest that cumin may promote weight loss through increased adiponectin levels and reduced leptin resistance, which may suppress appetite and enhance energy expenditure (15, 16).

Several randomized controlled trials (RCTs) have investigated the effects of *cumin* supplementation on adults with metabolic abnormalities. However, most included trials focused on individuals with T2DM, overweight/obesity, prediabetes, or NASH, with only a limited number of studies specifically enrolling rigorously defined MetS patients (15, 17, 18). This discrepancy introduces indirectness and internal inconsistency between the stated research question, eligibility criteria, included populations, and conclusions.

Nonetheless, there is inconsistency among trials investigating the effects of *cumin* on components of MetS (19, 20). Samani and Farrokhi (21) administered 25 mg of *cumin* per day for individuals with MetS. This study was accompanied with significant improvement in term of glycemic state and lipid profile (TG and HDL-C). While, some other studies failed to show significant changes in term of FBS following *cumin* supplementation (15, 22). Similarly, Kazemipoor et al. (37) administered *cumin* for 12 weeks for obese subjects and illustrated a significant reduction in WC level. Whereas, other study failed to show positive effects for *cumin* regarding WC improvement in pre-diabetic patients. Also, none of these studies performed a Grading of Recommendations Assessment, Development and Evaluation (GRADE) assessment, making it impossible to comment on the quality of the obtained evidence. Third, none of these studies have focused on patients with MetS. Therefore, this study aims to systematically evaluate the impact of *cumin* supplementation on MetS components in adults with metabolic disorders through a meta-analysis of RCTs, while assessing the quality of evidence using the GRADE approach. By integrating these methodologies, this research seeks to provide comprehensive insights into *cumin’s* efficacy in managing metabolic disorders and identify areas for future investigation.

## 2 Methods

### 2.1 Trial registration

The present systematic review and meta-analysis were carried out in accordance with the guidelines provided by the Preferred Reporting Items for Systematic Reviews and Meta-Analyses (PRISMA) and followed the methodology outlined in the Cochrane Handbook of Systematic Reviews of Interventions (23). The systematic review and meta-analysis was registered in advance in the International Prospective Register of Systematic Reviews (PROSPERO; ID: CRD42024425894).

### 2.2 Search strategy

A thorough search of electronic databases including Web of Science, Scopus, Embase, and PubMed was conducted to identify relevant articles. Two reviewers independently screened and selected published articles up to April 2025. The search was restricted to articles published in English and studies involving human participants. There was no limit on publication dates. The detailed search strategy for each database is provided in the Supplementary material. Reference lists of included studies were also screened to identify additional relevant articles.

### 2.3 Eligibility criteria

Studies were eligible for inclusion if they met the following PICO (population, intervention, comparison, and outcome) criteria: (1) participants of adult ages (>18 years) with MetS; (2) for intervention, studies using *cumin*; (3) for comparison, studies examining the effects of *cumin* supplements versus a control or placebo group; and (4) for outcomes, studies that reported FBS, TG, HDL-C, and WC measured using a fully validated method. Nevertheless, studies were excluded if they: (1) did not include a control group; (2) were observational (case–control or cross-sectional), animal, or review studies; or (3) lacked adequate data or contained duplicate data.

### 2.4 Study selection

After eliminating duplicate records, the titles and abstracts of the remaining articles were evaluated independently by two reviewers to assess their relevance. Subsequently, the full texts of potentially eligible studies were thoroughly examined by the same reviewers to determine their eligibility for inclusion in the systematic review. In the event of any disagreements, resolutions were determined through discussions involving another author. The kappa coefficient measures the agreement between two reviewers, and in this study, the kappa statistic was approximately 0.8. Study selection was independently conducted by two reviewers.

### 2.5 Data extraction

The extracted study characteristics included the following: participant details such as biological sex, age, health conditions, and sample size; information regarding the dose of *cumin* used and the duration of the interventions in weeks. To analyze each outcome (FBS, TG, HDL-C, and WC), the pre- and post-intervention means and standard deviations, or mean differences and their corresponding standard deviations, were extracted. These data were then utilized in the meta-analyses to generate forest plots. In cases of missing or incomplete data, attempts were made to contact study authors for clarification. When studies reported medians and interquartile ranges (IQR), means were approximated by the median, and standard deviations (SD) were estimated by dividing the IQR by 1.35. If SDs were not reported but standard errors (SE) or confidence intervals (CI) were available, SDs were calculated as SD = SE × √*n* or, for 95% CIs, as SD = (upper CI limit − lower CI limit)/(2 × 1.96). For studies reporting only ranges, SDs were approximated by dividing the range by 4, assuming a normal distribution (24–26).

### 2.6 Quality evaluation and meta-evidence

The risk of bias was evaluated using the Cochrane RoB 2 tool to evaluate the quality of the included studies (27). This scale consists of five items: randomization, deviations from intended interventions, missing outcome data, outcome measurement, and selective reporting, with each domain rated as low, some concerns, or high risk. The quality of evidence was assessed using the GRADE approach (28). Two researchers independently assessed the certainty of the evidence as high, moderate, low, or very low, and any disagreements were resolved through discussion or, when necessary, consultation with a senior researcher.

### 2.7 Statistical analyses

All analyses were conducted using Stata software-16 (Stat Corp, College Station, TX, USA). Standardized mean difference (SMD), and 95% confidence intervals (CIs) were calculated for variables that had the same measurement unit. Heterogeneity was evaluated by using the *I*^2^ statistic and *Q*-test. Significance was set at *I*^2^ values >50% or the *Q*-test with *p* < 0.1 (29). Pooled estimates were obtained using a random-effects model (DerSimonian–Laird method), given the potential for clinical and methodological heterogeneity across studies (30). To find probable sources of heterogeneity, subgroup analyses were performed according to the predefined variables, including the duration of the interventions, age, dose, and health conditions. Additionally, sensitivity analysis were performed by excluding each study one by one to assess their individual impact on the overall results (31). Due to fewer included studies than ten, Begg’s test was used to assess publication bias (32). Significant value was set as <0.05 in all analyses.

## 3 Results

### 3.1 Study selection and characteristics of the studies

**Figure 1:**
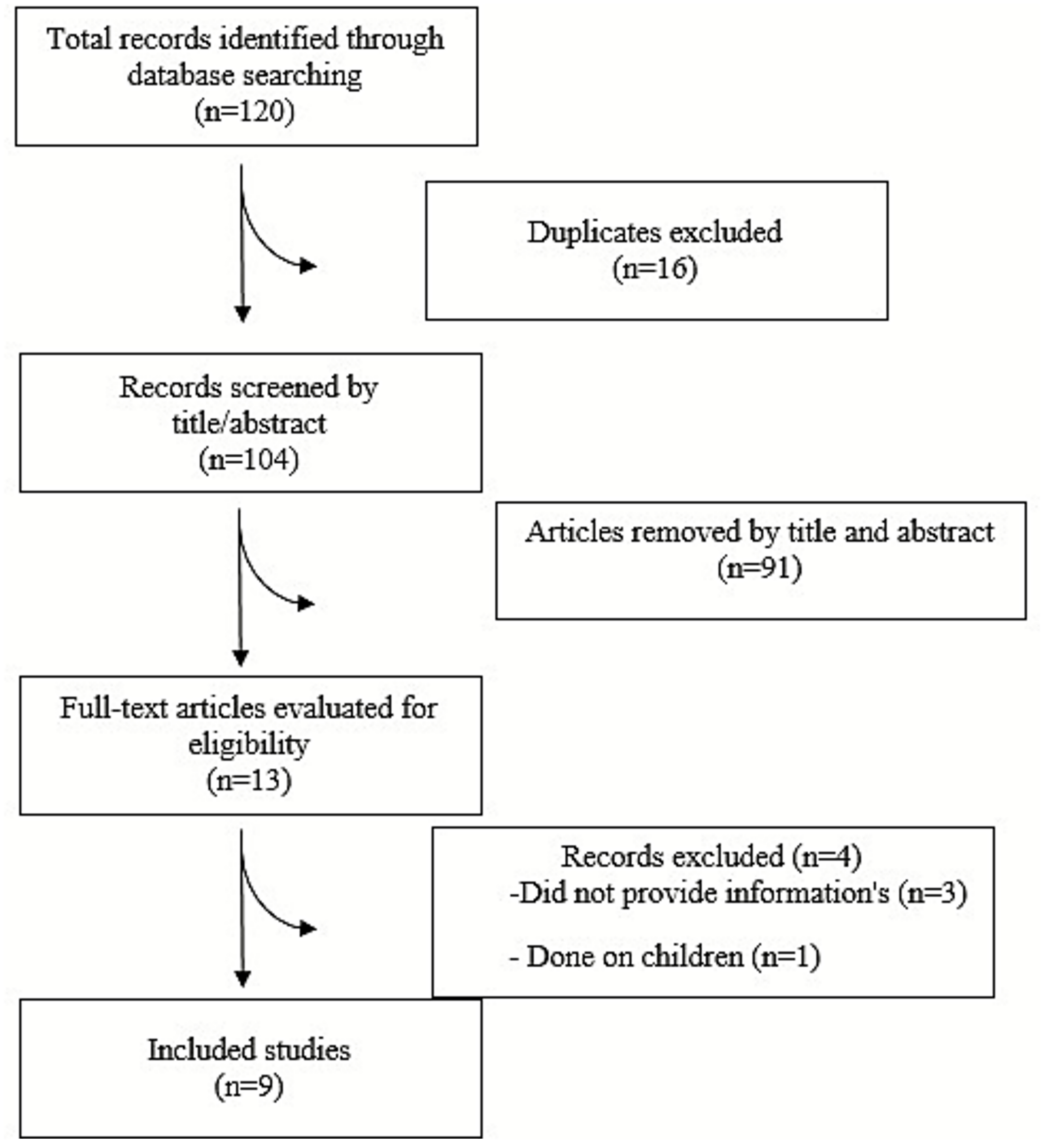
Flow diagram of study selection.

Our rigorous database searches uncovered 120 records, from which 104 were carefully selected after removing duplicates. Subsequently, 91 records were eliminated after the initial evaluation, leaving us with 13 records for full-text screening. During the second screening, four articles were excluded based on reasons detailed in Figure 1. Our comprehensive analysis included nine studies in the meta-analysis (Figure 1).

Based on the type of studied indices, the total number of included RCTs was as follows: eight for FBS, and TG; seven for HDL-C; and four for WC. A total sample size of 531 were included in current meta-analysis. Publications ranged from 2013 until 2020. Mean age of samples were ranged between 37 and 60 years. Administered *cumin* dosage in studies were between 0.025 g/day and 2 g/day in the form of oil or powder. Location of all included articles were in Iran (15, 17, 20, 22, 33–37). Table 1 provides a summary of the characteristics of the included studies.

**Table 1:** Study characteristics of included studies.

Author, year, country	Study design (blinding)	Location	Participants, n	Health condition	Duration (week)	Age, year	Baseline BMI (kg/m^2^)	Intervention/control	Quality of study
Morovati et al. (18, 22), (2019)	Parallel, RCT (triple)	Iran	M/F: 44	Metabolic syndrome	8	41	30	225 mg/day of cumin essential oil/placebo	High
Samani Keihan et al. (33) (2016)	Parallel, RCT (double)	Iran	M/F: 63	T2DM	12	59	29	25 mg/day of cumin essential oil/placebo	Low
Jafari et al. (17) (2018)	Parallel, RCT (double)	Iran	M/F: 45	T2DM	8	47	27	100 mg/day of cumin essential oil/placebo	High
Jafari et al. (17) (2018)	Parallel, RCT (double)	Iran	M/F: 54	Pre-diabetes	10	49	31	75 mg/day of cumin essential oil/placebo	High
Froghi et al. (35) (2018)	Parallel, RCT (double)	Iran	M/F: 80	T2DM	8	55	27	75 mg/day of cumin essential oil/placebo	Low
Taghizadeh et al. (15) (2015)	Parallel, RCT (double)	Iran	M/F: 52	Overweight subjects	8	37	31	300 mg/day of cumin essential oil/placebo	Low
Shavakhi et al. (20) (2015)	Parallel, RCT (double)	Iran	M/F: 81	NASH	24	38	30	75 mg/day of cumin essential oil/placebo	High
Kazemipoor et al. (37) (2013)	Parallel, RCT (triple)	Iran	F: 60	Obesity	12	50	30	30 mL/day of cumin essential oil/placebo	Low
Jafari-Maskouni et al. (36) (2020)	Parallel, RCT (double)	Iran	M/F: 52	T2DM	8	52	29	2000 mg/day of cumin essential oil/placebo	High

BMI, body mass index; RCT, randomized controlled trial; M, male; F, female; T2DM, type 2 diabetes mellitus; NASH, nonalcoholic steatohepatitis.

### 3.2 Risk of bias and grade assessment

Table 1 provides the results of the quality assessments of the included studies. Out of the nine RCTs included in the present review, five RCTs were of high quality. Using the GRADE approach, FBS, HDL-C, and TG outcomes were rated low, and WC was rated moderate for evidence quality (Table 2).

**Table 2:** Summary of findings and quality of evidence assessment using the GRADE approach.

Glycemic measures	Summary of findings		Quality of evidence assessment (GRADE)					
	No of patients (trials)	SMD (95% CI)	Risk of bias^a^	Inconsistency^b^	Indirectness^c^	Imprecision^d^	Publication bias^e^	Quality of evidence^f^
FBS	489 (8)	−1.38 (−2.26, −0.50)	Not serious	Serious	Serious	Not serious	Not serious	Low
TG	486 (8)	−0.58 (−1.14, −0.02)	Not serious	Serious	Serious	Not serious	Not serious	Low
HDL-C	416 (7)	0.77 (0.02, 1.52)	Not serious	Serious	Serious	Not serious	Not serious	Low
WC	210 (4)	−0.46 (−0.87, −0.04)	Not serious	Not serious	Serious	Not serious	Not serious	Moderate

FBS, fasting blood sugar; TG, triglyceride; HDL-C, high-density lipoprotein-cholesterol; WC, waist circumference.

a Risk of bias based on the Cochrane risk of bias tool. This tool assesses selection bias, performance bias, detection bias, attrition bias, and reporting bias. Five of eight included studies had incomplete outcome data (attrition bias). Half of included studies had performance bias.

b Downgraded if there was a substantial unexplained heterogeneity (*I*^2^ > 50%, *p* < 0.10) that was unexplained by meta-regression or subgroup analyses.

c Downgraded if there were factors present relating to the participants, interventions, or outcomes that limited the generalizability of the results.

d Downgraded if the 95% confidence interval (95% CI) crossed the minimally important difference (MID) for benefit or harm.

e Downgraded if there was an evidence of publication bias using funnel plot that affected overall results detecting by trim and fill analysis.

f Since all included studies were randomized controlled trials, the certainty of the evidence was graded as high for all outcomes by default and then downgraded based on prespecified criteria. Quality was graded as high, moderate, low, very low.

### 3.3 Effect of cumin supplementation on FBS

**Figure 2:**
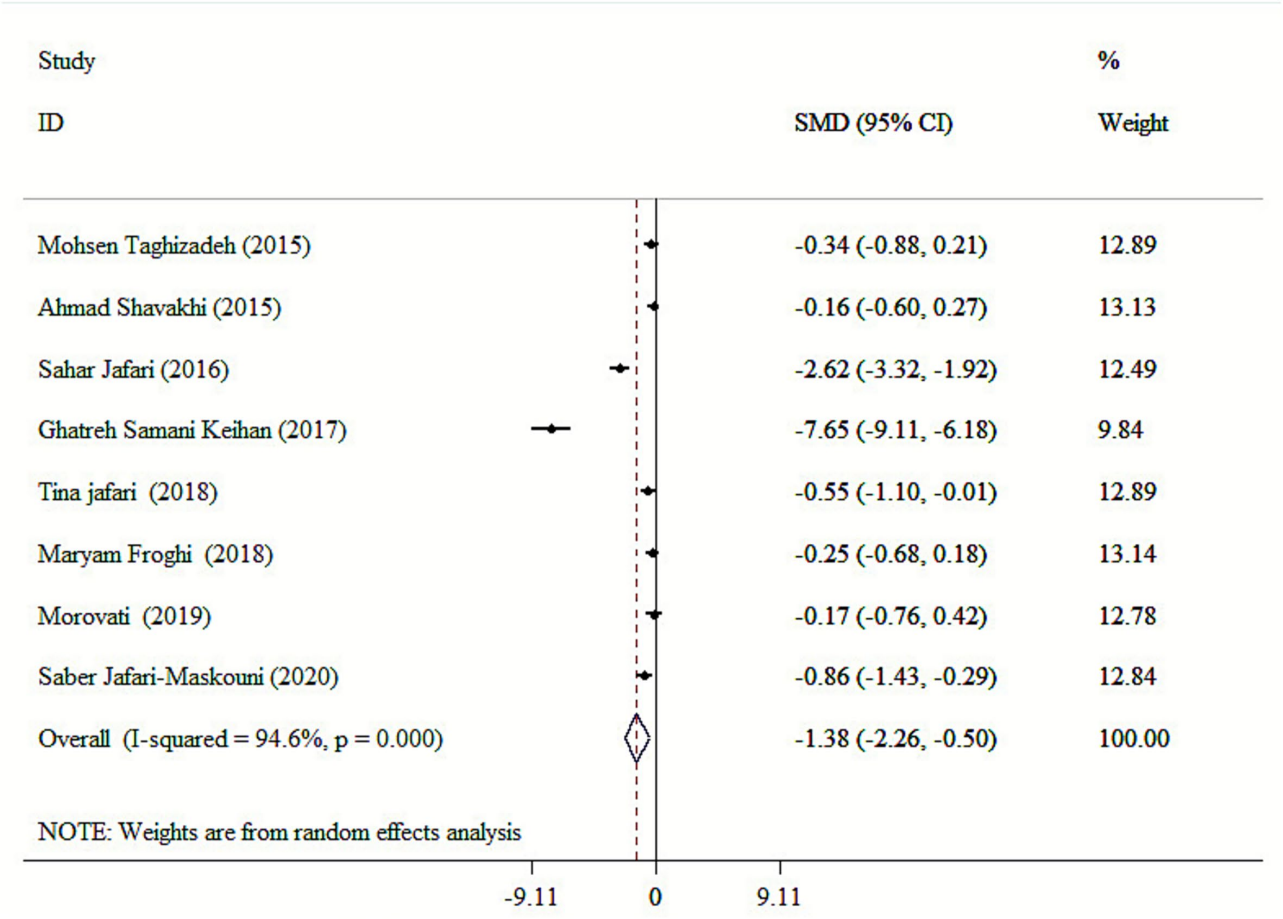
Forest plot for the effects of cumin supplementation on FBS levels.

Our finding revealed that *cumin* had a significant effect on FBS (SMD: −1.38; 95% CI: −2.26 to −0.50, *p* = 0.002; *I*^2^ = 94.6%, *p* < 0.001) (Figure 2). Mean age, health condition, dosage and duration were recognized as sources of high heterogeneity following subgroup analysis (Table 3). Performing subgroup analysis showed that the effect of *cumin* supplementation in dosage <100 mg/day on FBS in studies with an intervention duration of >8 weeks, a mean age >50 years, and subjects with T2DM were more robust than the entire sample (Table 3). Sensitivity analysis confirmed the robustness of the findings, with no single study exerting undue influence on the overall results. No significant publication bias was found in the Begg’s tests (*p* > 0.05).

**Table 3:** Subgroup analyses for the effects of cumin supplementation on components of metabolic syndrome.

Outcomes	Number of studies	SMD (95% CI)	*p*-within	*I*^2^ (%)	*p*-heterogeneity
*Cumin* on FBS					
Overall	8	−1.38 (−2.26, −0.50)	0.0002	94.6	<0.001
Age (year)					
≤50	5	−0.74 (−1.51, 0.03)	0.059	89.7	<0.001
>50	3	−2.77 (−5.36, −0.19)	0.036	97.8	<0.001
Dose (mg/day)					
<100	4	−1.91 (−3.50, −0.33)	0.018	96.8	<0.001
≥100	4	−0.98 (−1.97, 0.01)	0.053	90.9	<0.001
Intervention duration (week)					
≤8	5	−0.82 (−1.59, −0.05)	0.036	89.5	<0.001
>8	3	−2.64 (−5.21, −0.07)	0.044	97.8	<0.001
Health conditions					
T2DM	5	−2.22 (−3.71, −0.73)	0.003	96.5	<0.001
NASH	1	−0.16 (−0.60, 0.27)	0.464	–	–
Obesity	1	−0.34 (−0.88, 0.21)	0.229	–	–
MetS	1	−0.17 (−0.76, 0.42)	0.575	–	–
*Cumin* on HDL-C levels					
Overall	7	0.77 (0.02, 1.52)	0.045	92.3	<0.001
Age (year)					
≤50	5	0.50 (−0.01, 1.01)	0.055	78.6	<0.001
>50	2	1.56 (−2.05, 5.17)	0.397	98.2	<0.001
Dose (mg/day)					
<100	4	1.43 (0.36, 2.50)	0.009	93.2	<0.001
≥100	3	−0.10 (−0.43, 0.22)	0.528	0.0	0.657
Intervention duration (week)					
≤8	3	−0.10 (−0.43, 0.22)	0.528	0.0	0.657
>8	4	1.43 (0.36, 2.50)	0.009	93.2	<0.001
Health conditions					
T2DM	3	1.31 (−0.60, 3.21)	0.178	96.5	<0.001
Obesity	2	0.13 (−0.31, 0.57)	0.562	33.1	0.221
Others	2	0.71 (−0.45, 1.86)	0.229	89.1	0.002
*Cumin* on TG					
*Overall*	8	−0.58 (−1.14, −0.02)	0.044	88.7	<0.001
Age (year)					
≤50	5	−0.10 (−0.35, 0.15)	0.431	12.6	0.334
>50	3	−1.37 (−2.69, −0.05)	0.042	93.8	<0.001
Dose (mg/day)					
<100	5	−0.78 (−1.68, 0.12)	0.089	93.3	<0.001
≥100	3	−0.27 (−0.59, 0.06)	0.107	0.0	0.858
Intervention duration (week)					
≤8	4	−0.44 (−0.76, −0.12)	0.007	31.7	0.222
>8	4	−0.77 (−1.95, 0.41)	0.199	94.8	<0.001
Health conditions					
T2DM	4	−1.15 (−2.11, −0.19)	0.019	91.5	<0.001
Obesity	2	−0.03 (−0.40, 0.35)	0.892	0.0	0.335
Others	2	0.02 (−0.33, 0.37)	0.898	0.0	0.415
*Cumin* on WC					
*Overall*	4	−0.46 (−0.87, −0.04)	0.033	56.1	0.077
Dose (mg/day)					
<100	2	−0.67 (−1.48, 0.14)	0.106	77.8	0.034
≥100	2	−0.23 (−0.64, 0.17)	0.254	0.0	0.974
Intervention duration (week)					
≤8	2	−0.23 (−0.64, 0.17)	0.254	0.0	0.974
>8	2	−0.67 (−1.48, 0.14)	0.106	77.8	0.034

FBS, fasting blood sugar; TG, triglyceride; HDL-C, high-density lipoprotein-cholesterol; WC, waist circumference; T2DM, type 2 diabetes mellitus; NASH, nonalcoholic steatohepatitis.

### 3.4 Effect of cumin supplementation on TG

**Figure 3:**
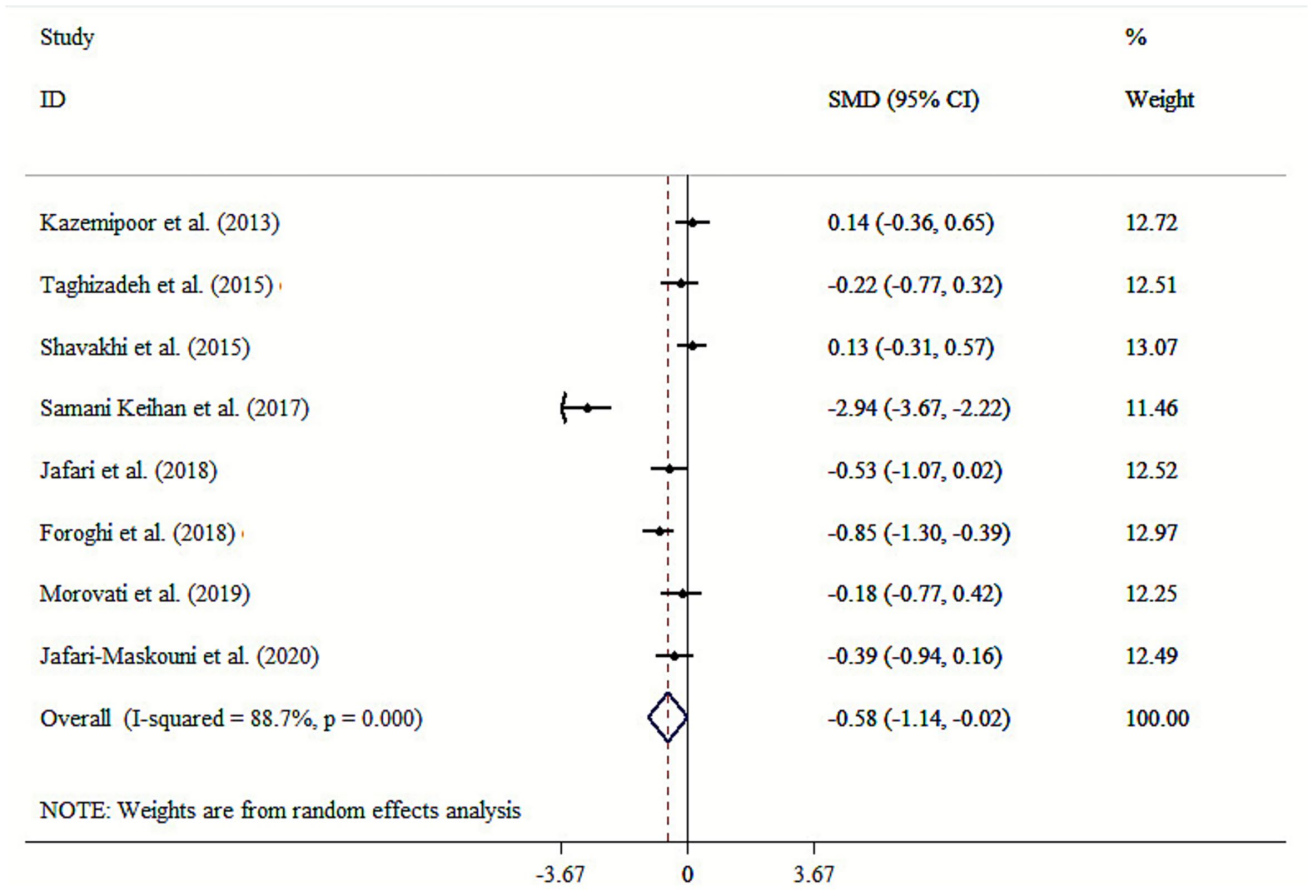
Forest plot for the effects of cumin supplementation on TG levels.

The results of our analysis indicated that *cumin* supplementation substantially decreased TG levels (SMD: −0.58; 95% CI: −1.14, −0.02, *p* = 0.044; *I*^2^ = 88.7%, *p* < 0.001) (Figure 3). Subgroup analysis identified mean age, health status, dosage, and intervention duration as source contributors to the observed heterogeneity (Table 3). Subgroup analysis indicated that *cumin* supplementation with an intervention duration of ≤8 weeks, a mean age >50, and in patients with T2DM contributes to a greater effect in lowering TG (Table 3). The sensitivity analysis demonstrated strong and consistent findings, with no individual study significantly impacting the results. Begg’s tests found no significant publication bias (*p* > 0.05).

### 3.5 Effect of cumin supplementation on HDL-C

**Figure 4:**
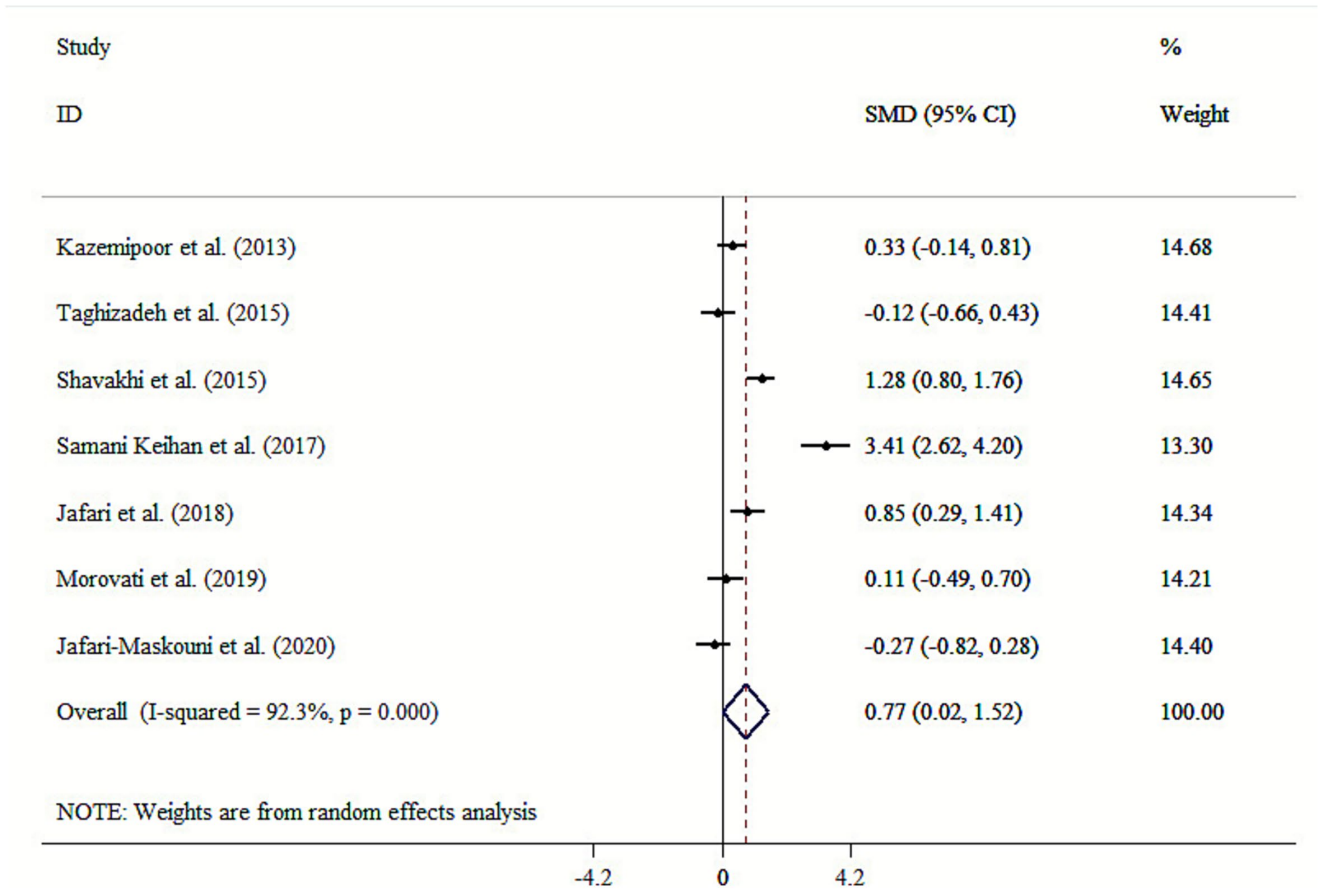
Forest plot for the effects of cumin supplementation on HDL-C levels.

Our finding revealed that *cumin* had a significant effect on HDL-C level (SMD: 0.77; 95% CI: 0.02 to 1.52, *p* = 0.045; *I*^2^ = 92.3%, *p* < 0.001) (Figure 4). Subgroup analyses revealed that mean age, health condition, intervention dosage, and duration were significant sources of heterogeneity (Table 3). Performing subgroup analysis showed that the effect of *cumin* supplementation in dosage <100 mg/day on HDL-C in studies with an intervention duration of >8 weeks were more robust than the entire sample (Table 3). Sensitivity analysis confirmed the robustness of the findings, with no particular study exerting undue influence on the overall results. Begg’s test did not show any significant publication bias (*p* > 0.05).

### 3.6 Effect of cumin supplementation on WC

**Figure 5:**
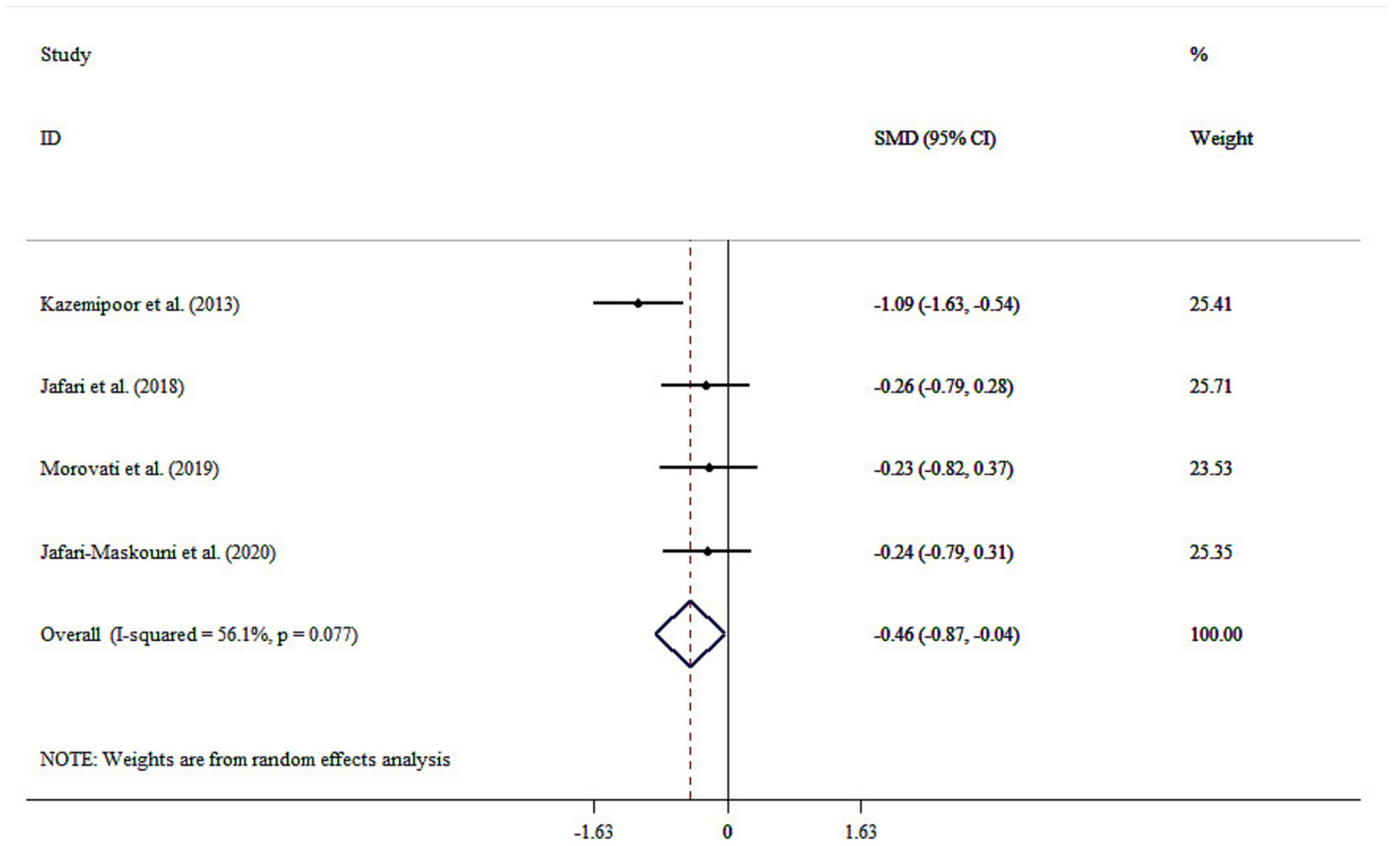
Forest plot for the effects of cumin supplementation on WC levels.

The results of our analysis indicated that *cumin* supplementation substantially reduced WC levels (SMD: −0.46; 95% CI: −0.87, −0.04, *p* = 0.033; *I*^2^ = 56.1%, *p* = 0.077) (Figure 5). Sensitivity analysis showed that no individual study significantly influenced the results. Begg’s tests found no significant publication bias (*p* > 0.05).

## 4 Discussion

The current meta-analysis summarized the results of nine RCTs involving 531 participants. It demonstrated that *cumin* supplementation may exert beneficial effects on the management of metabolic syndrome. Subgroup analyses revealed that age, baseline health condition, intervention dosage, and duration were key sources of heterogeneity among the included studies. It is important to note that the subgroup findings (e.g., age >50 years, dose <100 mg/day, duration >8 weeks, T2DM) are exploratory and hypothesis-generating rather than definitive. Given the small number of studies per subgroup and persistent heterogeneity, these results should not be interpreted as prescriptive clinical recommendations. The observed trends in specific subgroups may suggest potential mechanisms or populations that could benefit more from cumin supplementation, but further high-quality trials are needed to confirm these findings. For instance, participants >50 years exhibited a greater reduction in FBS levels compared to younger individuals, possibly due to the increased prevalence of insulin resistance in older adults. This supports the hypothesis that *cumin* supplementation may exert more pronounced effects in populations with impaired glucose metabolism. Additionally, participants with T2DM showed significantly greater improvements in FBS, further reinforcing the relevance of *Cuminum cyminum L*. as a potential adjunctive therapy in this subgroup. Interestingly, a significant hypoglycemic effect was also observed at lower doses (<100 mg/day), suggesting that even minimal supplementation may yield metabolic benefits. Nonetheless, given the high heterogeneity in both subgroups, it is crucial to interpret these findings cautiously. These findings highlight the importance of considering participant characteristics and intervention parameters when interpreting the effects of *cumin* supplementation, and they underscore the need for more stratified analyses in future trials. This finding suggests that the bioactive compounds in cumin may exert their effects through modulation of cellular signaling pathways at low concentrations, potentially via mechanisms such as enhanced insulin sensitivity. Nonetheless, given the high heterogeneity in both subgroups, it is crucial to interpret these findings cautiously. The antidiabetic effects of *cumin* are attributed to its aldehyde compounds, especially *cumin* aldehyde, which can inhibit alpha-glucosidase and thus reduce glucose absorption in the small intestine (38, 39). In addition, *cumin* aldehyde can inhibit the function of aldose reductase, which increases the production of reactive oxygen species (ROS) in tissues (39). Through its antioxidant compounds, *cumin* reduces serum levels of tumor necrosis factor-α, a pro-inflammatory cytokine that inhibits insulin signaling and its biological actions, which can improve insulin sensitivity (40, 41). Notably, Jafari et al. (17) found that a 100 mg dosage of cumin resulted in a significantly greater reduction of FBS compared to a 50 mg dosage. However, both short- and long-term treatment showed significant effect on FBS. Likewise, it is important to pay attention to doses of *cumin* supplementation irrespective of treatment duration. Literature review showed supportive studies in line with our findings to improve components of MetS in patients with T2DM (33, 36, 42). While, several studies reported contradictory results in this regard (20, 22). It may be attributed to various *cumin* preparation, dosage, study population and severity of health condition.

In addition, *cumin* had ameliorative effects on TG level too. Moreover, subgroup analysis showed significant reduction in older adults (>50 years old) as expected. Due to significant metabolic changes associated with aging, older adults often need more carefully adjusted and targeted therapeutic interventions to achieve desired treatment effects. Short-term treatment demonstrated beneficial effects in reducing TG levels. However, this improvement may reflect transient metabolic adaptation, as long-term interventions could lead to different outcomes. Bioactive compounds found in *cumin* may activate the peroxisome proliferator-activated receptors (PPARs) pathways. PPAR-α activation promotes fatty acid oxidation while suppressing TG synthesis in hepatocytes, contributing to reduced hepatic lipid accumulation (15, 43). Also, T2DM patients may experience some degree of dyslipidemia. In that case, the lipid-lowering effect of *cumin* may be pronounced in T2DM patients based on subgroup analysis. In this context, 10-week administration of *cumin* essential oil improved lipid profile except total cholesterol level in prediabetes patients (17).

Furthermore, the present study demonstrated that *cumin* increased HDL-C level significantly. However, this significant effect was most pronounced at lower doses and with long-term intervention, suggesting a dose- and duration-dependent interaction in *cumin*’s modulation of lipid metabolism (21). The HDL-C raising effect of cumin, especially at lower doses over longer periods, is indicating that formation of HDL-C through LCAT pathway require longer periods of metabolic adjustment. *Cumin* is rich in polyphenols, which have antioxidant properties. These properties of *cumin* may protect HDL-C from oxidation (19, 21). A possible mechanism for the hypocholesterolemic effects of *cumin* may be related to the stimulation of cholesterol 7 alpha hydroxylase in the liver, which converts cholesterol to bile acids. And the presence of saponin in *cumin*, which interferes with the liver-intestinal circulation of cholesterol, reduces its absorption. Also, these results may be explained by the possible role of *cumin* in regulating the action of β-hydroxy-beta-methylglutaryl-CoA reductase, which is the main enzyme in cholesterol synthesis (44, 45). In addition, *cumin* has a significant amount of certain phytosterols that reduce the absorption of cholesterol (46). Finally, the antioxidant content of cumin, e.g., vitamin C, carotenoids, vitamin E, and polyphenols can moderate LDL oxidation and reduce LDL-C levels (21, 47). Consistent with our finding, Jafarnejad et al. (42) demonstrated that *cumin* significantly affected the HDL-C level in overweight and T2DM patients.

On the other hand, *cumin* supplementation was associated with a significant reduction in WC, indicating its potential in addressing central obesity. As mentioned previously, the antioxidant and anti-inflammatory properties of cumin play a key role in triggering the reduction in WC, likely by mitigating oxidative stress and inflammation, which are major contributors to abdominal fat accumulation (40).

It is important to highlight that while this meta-analysis demonstrates the potential of *cumin* supplementation in improving metabolic outcomes, substantial heterogeneity was observed across the included studies. Therefore, the interpretation of subgroup findings should remain cautious, and the results should be considered exploratory rather than definitive. This variability in results may be attributed to both methodological and biological factors. Notably, differences in *cumin* formulations and dosages appear to be key contributors to this heterogeneity. To address this issue, a subgroup analysis was conducted. However, it is recommended that future studies be performed with larger, more homogeneous populations to enhance the reliability and generalizability of the findings. Therefore, future RCTs employing varied dosages and assessment approaches across diverse regions are needed to strengthen and broaden the evidence base.

The quality of obtained evidence for outcomes was moderate to high. Although, there was a considerable risk of bias in some included studies, both high and low-qualified studies approved beneficial effects of cumin. Furthermore, the use of the GRADE approach in our study allowed for a more rigorous evaluation of the evidence quality, which evidence rated moderate to high. Registered protocol in PROSPERO, adherence to PRISMA guidelines, performing comprehensive subgroup analyses, low risk of publication bias, and grade approach were the strengths of our study. The novelty of this study lies in its focused examination of the effects of cumin specifically within MetS populations, distinguishing it from prior research. Our meta-analysis study has limitations that should be noted. First, due to the limited number of studies, subgroup analysis was not possible for WC. Secondly, in most of the included studies, the duration of supplement use was short. In addition, most of the included studies had incomplete outcome data. Therefore, further long-term clinical trials focusing on different forms of cumin are needed to clarify this issue. All studies included in this review were conducted in Iran, which may limit the generalizability of the findings. Variations in dietary habits, genetic backgrounds, and clinical management of metabolic syndrome within the Iranian population could affect the observed effects of *Cuminum cyminum L*. supplementation and may not reflect outcomes in other populations. Therefore, this geographical focus is considered a limitation of our study. To enhance the external validity of these results, future randomized controlled trials from diverse regions, such as Asia, Europe, and North America, are needed to evaluate the cross-cultural applicability of the findings. Due to the very limited number of studies reporting BP, this core component of MetS could not be included in the meta-analysis, which limits the comprehensiveness of the findings. In addition, smaller number of studies were included for each outcome, which suggests that funnel plots and Begg/Egger tests may present insufficient power. Accordingly, the findings should be interpreted with cation.

## 5 Conclusion

*Cumin* has improved and had favorable effects on components of MetS. Long-term (>8 weeks) supplementation *cumin* (<100 mg/day) in the age of more than 50 years is a more efficacious approach in this review. According to the present study, *cumin* can be used as an effective adjuvant agent, particularly for patients with T2DM. Due to the limitations of our research, it is essential to interpret the results with caution.

## Data Availability

The datasets presented in this study can be found in online repositories. The names of the repository/repositories and accession number(s) can be found in the article/Supplementary material.

## Glossary

MetS: metabolic syndrome
NCEP-ATP III: National Cholesterol Education Program Adult Treatment Panel III
IDF: International Diabetes Federation
FBS: fasting blood sugar
BP: blood pressure
TG: triglycerides
WC: waist circumference
HDL-C: high-density lipoprotein cholesterol
TNF-α: tumor necrosis factor-α
IL-6: interleukin-6
RCTs: randomized controlled trials
TG: triglyceride
GRADE: Grading of Recommendations Assessment Development and Evaluation
PRISMA: Preferred Reporting Items for Systematic Reviews and Meta-Analyses
IQR: interquartile ranges
SD: standard deviations
SE: standard errors
CI: confidence intervals
SMD: standardized mean difference
ROS: reactive oxygen species
PPARs: peroxisome proliferator-activated receptors
