# Supplementary material for: Potential benefits of Cuminum cyminum L supplementation on components of metabolic syndrome in adults with metabolic disorders: a GRADE-assessed systematic review and meta-analysis of randomized controlled trials
Source: Front Nutr. 2026 Jan 13;12:1618108. doi: 10.3389/fnut.2025.1618108 (PMC12834730; doi:10.3389/fnut.2025.1618108)
Supplement: Supplementary file 1 [file Table_1.DOCX]

**Search strategy for electronic databases**

| **Database** | **Search String** |  |
| --- | --- | --- |
| **PubMed** | ("cuminum"[MeSH Terms] OR "cuminum"[Title/Abstract] OR "cumin"[Title/Abstract] OR "cuminum cyminum"[Title/Abstract] OR "bunium persicum"[Title/Abstract] OR "black caraway"[Title/Abstract]) AND ("body mass index"[MeSH Terms] OR "waist circumference"[MeSH Terms] OR "body weight"[MeSH Terms] OR "blood glucose"[MeSH Terms] OR "glucose"[Title/Abstract] OR "anthropometry"[MeSH Terms] OR "FBS"[Title/Abstract] OR "lipids"[MeSH Terms] OR "total cholesterol"[Title/Abstract] OR "TC"[Title/Abstract] OR "triglyceride"[Title/Abstract] OR "TG"[Title/Abstract] OR "high-density lipoprotein cholesterol"[Title/Abstract] OR "HDL-C"[Title/Abstract] OR "BMI"[Title/Abstract]) AND ("randomized controlled trial"[Publication Type] OR "clinical trial"[Publication Type] OR "intervention"[Title/Abstract] OR "trial"[Title/Abstract]) |  |
| **Scopus** | (TITLE-ABS-KEY("cuminum" OR "cumin" OR "cuminum cyminum" OR "bunium persicum" OR "black caraway")) AND (TITLE-ABS-KEY("body mass index" OR "waist circumference" OR "body weight" OR "glucose" OR "FBS" OR "lipids" OR "total cholesterol" OR "TC" OR "triglyceride" OR "TG" OR "high-density lipoprotein cholesterol" OR "HDL-C" OR "BMI")) AND (TITLE-ABS-KEY("randomized" OR "randomised" OR "clinical trial" OR "intervention")) |  |
| **Embase** | ('cuminum'/exp OR 'cuminum' OR 'cumin' OR 'cuminum cyminum' OR 'bunium persicum' OR 'black caraway') AND ('body mass index'/exp OR 'waist circumference'/exp OR 'body weight'/exp OR 'glucose'/exp OR 'FBS' OR 'lipids'/exp OR 'total cholesterol' OR 'TC' OR 'triglyceride' OR 'TG' OR 'high-density lipoprotein cholesterol' OR 'HDL-C' OR 'BMI') AND ('randomized controlled trial'/exp OR 'clinical trial'/exp OR 'intervention' OR 'trial') |  |
| **Web of Science** | (TS=("cuminum" OR "cumin" OR "cuminum cyminum" OR "bunium persicum" OR "black caraway")) AND (TS=("body mass index" OR "waist circumference" OR "body weight" OR "glucose" OR "FBS" OR "lipids" OR "total cholesterol" OR "TC" OR "triglyceride" OR "TG" OR "high-density lipoprotein cholesterol" OR "HDL-C" OR "BMI")) AND (TS=("randomized" OR "randomised" OR "clinical trial" OR "intervention" OR "trial")) |  |
